# The Toxicological Impacts of the *Fusarium* Mycotoxin, Deoxynivalenol, in Poultry Flocks with Special Reference to Immunotoxicity

**DOI:** 10.3390/toxins5050912

**Published:** 2013-04-29

**Authors:** Wageha Awad, Khaled Ghareeb, Josef Böhm, Jürgen Zentek

**Affiliations:** 1 Clinic for Avian, Reptile and Fish Medicine, University of Veterinary Medicine, Veterinärplatz 1, Vienna A-1210, Austria; E-Mail: wageha.awad@vetmeduni.ac.at; 2 Institute of Animal Nutrition and Functional Plant Compounds, Department for Farm Animals and Veterinary Public Health, University of Veterinary Medicine, Veterinärplatz 1, Vienna A-1210, Austria; E-Mails: khaled.ghareeb@vetmeduni.ac.at (K.G.); josef.boehm@vetmeduni.ac.at (J.B.); 3 Department of Animal hygiene, Behavior and Management, Faculty of Veterinary Medicine, South Valley University, Qena 83523, Egypt; 4 Institute of Animal Nutrition, Department of Veterinary Medicine, Freie Universität Berlin, Königin-Luise-Str, 49, Berlin D-14195, Germany

**Keywords:** deoxynivalenol, *Fusarium* mycotoxin, immune responses, gut immunity, cytokines, poultry

## Abstract

Deoxynivalenol (DON) is a common *Fusarium* toxin in poultry feed. Chickens are more resistant to the adverse impacts of deoxynivalenol (DON) compared to other species. In general, the acute form of DON mycotoxicosis rarely occurs in poultry flocks under normal conditions. However, if diets contain low levels of DON (less than 5 mg DON/kg diet), lower productivity, impaired immunity and higher susceptibility to infectious diseases can occur. The molecular mechanism of action of DON has not been completely understood. A significant influence of DON in chickens is the impairment of immunological functions. It was known that low doses of DON elevated the serum IgA levels and affected both cell-mediated and humoral immunity in animals. DON is shown to suppress the antibody response to infectious bronchitis vaccine (IBV) and to Newcastle disease virus (NDV) in broilers (10 mg DON/kg feed) and laying hens (3.5 to 14 mg of DON/kg feed), respectively. Moreover, DON (10 mg DON/kg feed) decreased tumor necrosis factor alpha (TNF-α) in the plasma of broilers. DON can severely affect the immune system and, due to its negative impact on performance and productivity, can eventually result in high economic losses to poultry producers. The present review highlights the impacts of DON intoxication on cell mediated immunity, humoral immunity, gut immunity, immune organs and pro-inflammatory cytokines in chickens.

## 1. Introduction

**Figure 1:**
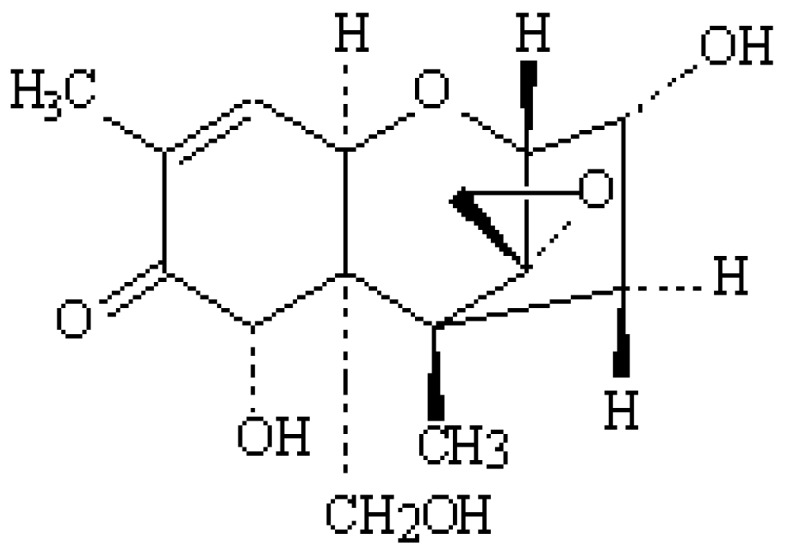
Chemical structure of deoxynivalenol.

*Fusarium* mycotoxins frequently contaminate cereal grains, which are the main constituents of poultry feeds. Deoxynivalenol (DON) is a mycotoxin produced by *Fusarium* species. It is considered as one of the most important trichothecenes and found in all kinds of grains, such as wheat, rye, barley and oats [1]. The chemical structure of DON (Figure 1) is stable and resists low pH levels, and it consequently can contaminate the diets of humans and animals, including poultry [2]. 

The adverse impacts of DON mycotoxin on immune function have been documented in experimental animals, pigs and poultry and cell culture models. However, it is not completely known, how DON modulates the immune responses. It seems likely that DON alters the viability and proliferation of immune cells. This, in turn, results in an inhibition of protein biosynthesis and alteration of the pro-inflammatory cytokine production [3,4,5].

The impact of DON on the immune system ranges from immunosuppression to immunostimulation, according to its concentration, duration and time of exposure [6,7,8]. Interestingly, low concentrations of DON (less than 5 mg/kg feed) seem to be responsible for a stimulation of immunity and high concentrations seem to suppress the immune responses [9]. Chronic DON intoxication at high concentrations leads to injuries of rapidly and actively dividing cells of immune organs and mucosa of the gastrointestinal tract [3]. As with other trichothecenes, protein biosynthesis is inhibited by DON. The toxin binds to the 60S subunit of ribosomes. This has been shown to induce a stress response and mitogen activated protein kinases (MAPKs) were activated, due to ribosomal conformational changes affecting the peptidyl transferase activity of ribosomes. After the induction of cyclooxygenase 2 (COX2), prostaglandin levels were elevated. An important activity of MAPKs is their effect on transcription factors. Higher expression of nuclear factor κB (NF-κB) induces the expression of proinflammatory cytokines affecting immune reactions in animals [10]. It was clearly known that the signs of DON intoxication in experimental animals can be explained by a dysregulation of pathways relevant for cell signaling and an impact on genes having relevance for immunological reactions [11]. 

The literature regarding the impact of DON feeding on health and performance traits in poultry flocks is contrary [12]. However, the immune dysfunctions due to DON exposure can predispose the birds to infectious diseases [4]. DON was shown to suppress the antibody response to infectious bronchitis vaccine (IBV) and to Newcastle disease virus in broiler chickens and laying hens, respectively [13,14]. Furthermore, DON decreased the concentration of tumor necrosis factor alpha (TNF-α) in plasma of broiler chickens [15]. TNF-α is an important cytokine involved in systemic inflammation and stimulates the acute phase reaction. DON, therefore, can interfere with production of TNF-α from macrophages. The reduction of TNF-α in the plasma after chronic feeding of DON in this study is a significant indicator that DON can impair immune function and increase susceptibility to infectious diseases. In addition, DON adversely affected the intestinal histomorphology, electrophysiology, absorption and barrier function in chickens [16,17,18,19]. These toxic effects of DON on the immune response and on intestinal functions of chickens are summarized in this review as indicators for the adverse effects of DON on poultry health.

## 2. Occurrence of DON in Poultry Feed

Deoxynivalenol (DON) is also called vomitoxin and produced by *Fusarium graminearum* (*Gibberella zeae*) and *F. culmorum* [20]. DON is the most common contaminant of feedstuffs worldwide. It was found in cereal grains (wheat, maize, barley, oat and rye and less often in rice, sorghum and triticale). DON contaminates mainly corn and wheat, while small grains, such as oats, rye and barley, have less DON contamination [21]. *Fusarium graminearum* and *F. culmorum* can survive in the leaves of the cold season and be a source of infection for the new crop. 

Cool temperatures and high humidity are the environmental conditions that favor the fungal development in the field [1,22]. After harvest, fungal infection can also occur in grains in the case of improper storage conditions, such as high moisture. After infection of grains, *F. graminearum* resulted in the diseases known as ear rot in corn or head blight in wheat and barley [22]. 

Deoxynivalenol contamination can be noticed when corn kernels ripen prematurely and unevenly and have a blanched appearance. At harvest, kernels may show pink color. The natural occurrence of DON in grains used for poultry is normally between 0 and 5 mg/kg, although concentrations can be higher [1]. However, improved storage conditions (<14% moisture) will minimize further elaboration of DON.

## 3. Effects on Growth Performance

Contamination of animal diets with DON can result in great economic losses for animal production. In poultry, it was shown that the impact of DON on growth performance is highly variable (Table 1), due to differences in strains of poultry and diets used. Chickens are considered to be less sensitive compared to other species, especially the pig. This can be attributed to differences in DON absorption, distribution, metabolism and elimination [23], in addition to the hypothesis of a protective effect of the renal first pass effect, which exists in chickens [24]. The first pass effect is mediated by different gastrointestinal and hepatic enzymes that result in the oxidation, reduction or hydrolysis (phase I reactions) and/or conjugation (phase II reactions) of toxins. This metabolism of orally administered mycotoxin results in a significant reduction of the amount of un-metabolized DON reaching the systemic circulation. Chickens resist DON due to the low level of absorption into plasma and tissues in addition to rapid clearance [25]. A limited oral absorption and rapid plasma clearance of DON was found in turkeys [26]. The intestinal microflora can convert DON to de-epoxy DON (DOM-1) in birds [27,28]. Whereas the degradation of the epoxide group by reductive cleavage of the toxic 12, 13-epoxy ring is carried out by intestinal microflora in chickens. Awad *et al.* [16,17] indicated that the *Eubacterium* sp. DSM 11798 was capable of completely compensating for the adverse effect of DON in poultry. Furthermore, deoxynivalenol did not accumulate in tissues and eggs [28]. However, in the absence of clear clinical symptoms, DON can induce reduced feed intake, which cannot be separated from the direct effect of DON contamination itself on immunity, hematology and health parameters [24]. 

**Table 1:** Effects of deoxynivalenol on performance traits in poultry.

Deoxynivalenol (mg/kg diet) Birds (species and sex)	Duration of exposure (days)	Performance traits	Reference
Up to 15 mg/kg diet Broiler (male or mixed sex)	21–42	No effects on performance	[16,17,18,35,36,37,38,39,40,41]
10–15 mg/kg diet Broiler (male or mixed sex)	21–42	Reduced feed intake, weight gain and feed efficiency	[13,29,30,31]
20 mg/kg diet Turkey poults (female)	21	No effects on performance	[45]
2–3 mg/kg Laying hens (aged 48 weeks)	56	No effects on performance and egg production	[42]
0.02 mg/kg Lohman Brown laying hens	42	Reduced feed intake	[14]

A reduction in body weight, feed intake and body weight gain of broilers fed diets artificially contaminated with 10 mg DON/kg diets was documented by Ghareeb *et al.* and Awad *et al.* [13,29]. A linear reduction in the feed intake and a slight decrease in body weight gain of broiler chickens fed with a diet contaminated with *Fusarium* mycotoxins, including DON as a major toxin, were found by Dänicke *et al.* [30]. Feeding broilers with up to 18 mg of DON /kg reduced body weight gain at the third week of life [31]. In laying hens, performance traits were adversely affected by chronic feeding of DON [14]. Egg production was negatively affected in hens fed a diet containing sorghum that was contaminated with zearalenone (ZON) at a level of 1.1 mg/kg and DON at a level of 0.3 mg/kg [32]. The effect in this study was due to the synergistic effect of DON and ZON.

In Peking ducklings, feed refusal was observed after natural contamination of the diet with 0.3–1.2 mg DON/kg and 0.01 mg of aflatoxin B1/kg feed [33]. In turkeys, feeding of corn contaminated with DON up to 10 mg/kg reduced poults body weight gain at the third week of life [31]. Only a slight reduction in the body weight gain was found in turkeys when fed increasing proportions of *Fusarium* toxin-deoxynivalenol contaminated wheat (0.10, 1.96, 4.66 and 5.42 mg DON/kg diet) [34].

However, some studies failed to notice an adverse effect on performance of poultry, including broilers, laying hens, ducklings and turkeys. In broilers, even levels of DON up to 15 mg/kg could not produce an adverse influence on body-weight gain, feed intake or feed efficiency [16,17,18,35,36,37,38,39,40,41]. In addition, performance of laying hens, egg production, fertility and hatchability of eggs remained unaffected after feeding of 2–3 mg/kg DON [42]. It is reported that DON decreased the small intestinal absorption of glucose and amino acids in broilers and laying hens [16,43], which can displace the nutrient uptake to the intestinal distal parts. It was shown that chickens can absorb glucose and amino acids in the large intestine [44], whereas the absorptive functions may be protected from the negative effect of DON. It was reported that DON can be completely transformed to de-epoxy-DON after incubating for 96 h with the content of the large intestine of hens [27]. This may explain why DON did not strongly influence the performance traits in some studies regarding broilers, laying hens, ducklings and turkeys. 

## 4. Effects on the Immune System and Internal Organs

Deoxynivalenol is a common inhibitor of protein biosynthesis, binds to peptidyl transferase, inhibits the synthesis of RNA and DNA and alters cell membranes. Therefore, tissues of higher protein turnover, such as immune organs, liver and small intestine, are adversely altered by DON exposure [46]. For example, it was shown that feeding of Peking ducks with an increasing proportion of DON contaminated wheat (6–7 mg DON/kg and 0.05–0.06 mg ZON/kg) led to a relative decrease of the mass of the bursa of Fabricius [47], which may reduce the production of antibodies. Moreover, in ducks, higher heart, liver and pancreas weight were reported after feeding of DON [48], and in broilers gizzard, heart and bursa of Fabricius were having a higher weight [49,35]. On the other hand, the liver mass was reduced in broilers fed diets containing (9 or 18 mg DON/kg) [49]. 

Gizzard mucosa had small erosions in laying hens fed DON in a very high concentration of 82.8 mg/kg for about four weeks in addition to higher absolute and relative gizzard weights. This was considered as an irritant effect of DON on the mucosa as reported by Lun *et al.* [50]. In hens, a decrease of the weight of the small intestine was observed after *Fusarium* mycotoxin (0.02 DON and 0.002 mg ZON/kg) intake, such as Dänicke *et al.* [14]. 

## 5. Effects on Gut Health

The gastrointestinal tract (GIT) is considered as an important barrier against toxins and contaminants [51], and it has physical, chemical, immunological and microbiological characteristics. Intestines are large immune organs and have a broad capability for innate and acquired immune reactions against various antigens [52].

In broiler chickens, villus atrophy and alteration of villus crypts of broilers were found after feeding of either artificial or natural DON contaminated diets [16,17,40], and the structure of duodenal and jejunal mucosa was affected in the form of shorter and thinner villi due to DON exposure [41]. Those results suggest that DON adversely affects the intestinal digestive and absorptive functions. Contrary to that, in ducks [32] and in turkey poults [31,34,45], DON did not affect the intestinal histology.

The barrier function of the epithelial cell layer is essential for maintaining the mucosal integrity. It is mainly provided by tight and adherence junctions of the epithelial cells. Fungal toxins were known to affect the barrier function, as shown by Bouhet *et al.* [53]. The intestinal mucosal integrity can be assessed by different methods, for instance, via the measurement of trans-epithelial electrical resistance (TEER). DON affected the integrity of intestinal epithelium. The tight junction proteins, such as Zonula Occludens (ZO-1), occludin and claudin isoforms are required to maintain the intestinal epithelium integrity. Tight junction proteins decrease the transport of moderately small hydrophilic molecules by closing the luminal intercellular space. By this, they reduce and regulate the paracellular water and substrate transport. 

DON decreased the TEER of a human epithelial cell line in a dose of 10 µmol/L [54]. Jejunal resistance was elevated after DON exposure (10 µg/mL) in laying hens *in vitro*,as reported by Awad *et al.* [43]. The increased total tissue resistance after DON exposure can be attributed to a tighter epithelium and reduced paracellular permeability to ions. Based on these results, the higher trans-epithelial resistance was assumed to be due to the reduced trans-cellular ion transport [43]; however, further work would be necessary for a complete characterization. Recently, in Ussing chamber experiments, the duodenal tissue resistance (TEER) was lower in chickens receiving DON contaminated feed in a dose less than 5 mg/kg diet [55].

## 6. Effects on the Immune Responses

Deoxynivalenol has negative effects on growth, feed consumption and may induce intestinal alterations, neurological and reproductive problems [11]. However, the immune impairment is considered as the most important outcome of DON mycotoxicoses [6]. According to the dose, DON can stimulate or suppress the immune functions in both farm and experimental animals [6,24,43]. In general, high doses of DON alter different arms of the immune system. Decreased cell proliferation, higher apoptosis and necrosis of immune cells may explain negative effects on the cellular immune response; other findings indicate increased IgA production and higher susceptibility of animals against infections, as indicated by Pestka *et al.* [56]. Deoxynivalenol, like other trichothecenes in low doses, promoted the expression of several cytokines and chemokines [57,58]. Cytokine mRNA expression was shown to be changed and the reaction patterns included transcriptional and post-transcriptional mechanisms [11].

It was shown that DON can have both immunostimulatory and immunosuppressive effects according to concentration, time and duration of exposure [11]. They are immunotoxic at low dietary concentrations, even if there is no alteration of the productivity traits [6,24,43]. Studies of DON immunotoxicity have focused primarily on the mouse model, with few investigations on the possible effect in humans or domestic animals. Unfortunately, limited information is available regarding the immunotoxicity of DON in poultry. Therefore, information from other animal models is useful for scientists working in this area, allowing research focused on specific targets. 

### 6.1. Humoral Immunity

In chickens, humoral immunity can be either stimulated or impaired by DON and other trichothecenes. In poultry, serum antibody titers to common viral vaccines can be useful to evaluate the humoral immunotoxicity of DON [30]. The effects of DON on the antibody titers to common vaccines are reviewed in Table 2. For instance, in broiler chickens, DON was shown to suppress the vaccination response to infectious bronchitis virus (IBV) by Dänicke *et al.* [14] and to Newcastle disease virus (NDV) by Dänicke *et al.* and Harvey *et al.* [14,59]. Recently, DON was shown to suppress the antibody response to infectious bronchitis vaccine (IBV) in broiler chickens [13,60,61]. 

**Table 2:** Effects of deoxynivalenol on antibody titers to common vaccines in poultry. NDV, Newcastle disease virus; IBV, infectious bronchitis vaccine.

Deoxynivalenol (mg/kg diet) Birds (species and sex)	Duration of exposure (days)	Observed effects	Reference
Up to 12.21 mg/kg diet Broiler (male)	35	NDV increased at week 2 and 4 NDV decreased at week 5 IBV decreased at week 5 IBV not affected at week 2, 4, 5	[60]
10 mg/kg diet Broiler (male)	35	IBV decreased at week 5	[13]
3.5–14 mg/kg diet Broiler (male)	35	NDV decreased	[30]
4.7–8.3 mg/kg diet Broiler (male)	14	IBV not affected	[38]
12.6 mg/kg diet Broiler breeder	84	IBV decreased NDV not affected	[61]
18 mg/kg White leghorn chicks	72	NDV not affected	[59]
12.3 mg/kg Laying hens	112	NDV decreased	[14]

Furthermore, the feeding of contaminated diets with *Fusarium* mycotoxins to chickens did not cause significant changes in serum or bile immunoglobulin concentrations [5]. However, Swamy *et al.* [38] controversially observed that the feeding of contaminated grains with *Fusarium* mycotoxins caused significant linear and quadratic declines in the biliary IgA, but not in serum IgG and IgM. In contrast, IgA elevation is obtained as an immunopathological effect of induced cytokine upregulation in mice fed DON [62]. Higher values of serum IgA and IgE [63,64], stimulation of IgA secreting cells and poly-reactive IgA autoantibody secretion was also observed [65,66]. It was reported that the ability of DON to increase poly-clonally IgA-secreting cells can be related to the impact on macrophages and T-cells and their regulatory cytokine patterns *in vitro* [58,65]. Furthermore, a higher biliary IgA level was reported in turkey fed DON [67]. Contrary to this, it was shown that the biliary IgA was reduced by DON [38]. Recently, feeding a mixture of mycotoxins, including DON to broiler chickens, was shown to reduce IgA, the relative weight of the spleen, the mRNA expression of IFN-γ and the antibody titers against Newcastle disease [68]. 

### 6.2. Cellular Immunity

Deoxynivalenol in high doses resulted in apoptosis of leukocytes, which led to immunosuppression [11]. Apoptosis of T-cells, B-cells and IgA+ cells was found after DON exposure *in vitro* [69]. Such results are significant for poultry and other animal species, because the same effect was obtained in bone marrow, thymus and Peyer’s patches after trichothecene exposure in rodents [69]. Stimulation of macrophages by low doses of trichothecenes induced upregulation of the expression of several inflammation related genes, pro-inflammatory cytokines and several chemokines [70]. Contrary to this, exposure to *Fusarium* mycotoxins (T-2 toxin and DON) at higher levels was shown to increase apoptosis in macrophages and suppressed the innate immune function [11]. 

Dietary DON alters immune function in laying hens [67]. An important immunotoxic effect was seen after dietary inclusion of DON in diets for laying hens and broilers, such as the reduction of white blood cell and total lymphocyte numbers [67]. Deoxynivalenol produced genotoxic effects on circulating blood lymphocytes [29]. Leukocytes, isolated from chicken spleen, had higher DNA fragmentation when animals were exposed to 10 mg DON/kg feed [71]. Mechanistic studies have been rarely conducted; the activation of oxidative pathways, the formation of DNA adducts or DNA breaks are considered as important in broiler chickens. B-lymphocytes and CD4+ and CD8+ T-lymphocytes were decreased [70]. Furthermore, B-cells and T-cells were also decreased after DON feeding *in vivo* [5]. 

Chronic feeding of 10 mg DON/kg feed to broilers decreased the plasma concentration of TNF-α [15], which is an important cytokine released in case of inflammation. TNF-α is produced in large amounts in response to lipopolysaccharides (LPS) of bacterial and viral antigens, so that it is called LPS-induced TNF. The decreasing level of TNF-α is one of the possible impairment of immune function after chronic DON exposure in poultry and could thus increase the susceptibility to infectious diseases. However, this hypothesis needs further studies to be confirmed. 

## 7. Effects on Pro-inflammatory Cytokines

There is a lack of information about the influences of DON on cytokines in poultry. Most available studies were conducted on laboratory animals and on cell lines. DON in lower amounts stimulates the upregulation of cytokines and chemokines [72]. The efficacy of DON to alter the expression of immune relevant genes for cytokine synthesis *in vitro* includes transcriptional and posttranscriptional mechanisms [73], and protein biosynthesis is negatively affected. DON was shown to stimulate cytokine production and induce the abundance of IL-1 mRNA [74]. It was also shown that DON (lower than 25 ng/mL in the human U937 monocytic cell line) induced cytokine and chemokine expression in human blood monocytes *in vitro* [75]. Deoxynivalenol upregulated cytokine production in murine models *in vitro* and *in vivo* [76]. Other experiments indicated that mRNA expression in spleen and Peyer´s patches was elevated after DON exposure, including TNF-α, IL-1ß, IL-6, IL-12, interferon-γ (IFN-γ), IL-2, IL-4 and IL-10 [77]. Mice had increased levels of mRNA of IL-1ß, IL-6 and TNF-α, IFN-γ, IL-4 and IL-10 after a single oral dose of 5 and 25 mg/kg body weight [78]. It is noteworthy that the induction of different cytokines and chemokines could be the consequence of DON induced anorexia.

The capability of DON to affect the cytokine gene expression is significant information, because this can lead to a dysregulation of immune functions. In domestic pigs, lower IL-1β and IL-8 expressions occurred in blood and ileal tissue after feeding of low doses of DON [8]. Similarly, in broiler chickens, splenic mRNA expression of IFN-γ was downregulated as a result of chronic feeding of naturally contaminated diets with DON and other *Fusarium* mycotoxin contaminated diets [79]. Furthermore, plasma concentration of TNF-α was significantly reduced after chronic exposure to 10 mg DON/kg diet in 5 wk old broiler chickens [15]. In contrast, interferon-γ (IFN-γ) gene expression was upregulated in the caecal tonsils of chickens fed *Furarium* mycotoxins challenged with coccidia [80]. In this context, it becomes evident that further research is required to investigate the effects of DON on the innate immune response. 

## 8. Conclusions

Deoxynivalenol alters the health of poultry through its negative effects on gastrointestinal function and dysregulation of the immune system. Several studies showed significant chronic impacts of DON in poultry, including reduced feed intake, altered nutrient absorption and impaired immune responses. Furthermore, the negative impact of DON on the gut function and immune system together with other adverse toxic effects could be an important factor for increasing the susceptibility of poultry flocks to infectious diseases. Further research is required to investigate how DON alters signaling pathways, humoral and cellular reactions and the innate immune functions. Taking the current knowledge together, contamination of diets with DON has a high risk to adversely affect the production and health of chickens, and this might be especially relevant in an adverse rearing environment and management procedures.
